# Interpreting Transmissibility of COVID-19 in Children

**DOI:** 10.3201/eid2612.203452

**Published:** 2020-12

**Authors:** Eun Young Cho, Eun Hwa Choi, Jong-Hyun Kim

**Affiliations:** Chungnam National University Hospital, Daejeon, South Korea (E.Y. Cho);; Seoul National University College of Medicine, Seoul (E.H. Choi);; College of Medicine, The Catholic University of Korea, Seoul (J-H. Kim)

**Keywords:** coronavirus disease, SARS-CoV-2, severe acute respiratory syndrome coronavirus 2, severe acute respiratory syndrome, SARS, viruses, respiratory infections, zoonoses, COVID-19, SARS-related coronavirus, disease transmission, child, adolescent, schools

**To the Editor:** We read with great interest the article by Park et al. (*1*) on contact tracing of 5,706 patients with coronavirus disease (COVID-19) during the early phase of the pandemic in South Korea. In the study, the overall detection rate of COVID-19 among household contacts was 11.8%; the highest detection rate (18.6%) was in household contacts of those 10–19 years of age and the lowest detection rate (5.3%) in household contacts of those 0–9 years of age. The media have reported the research as evidence that transmissibility in adolescents and adults is similar (*2*). Such an interpretation may influence decision-making on the reopening of schools.

Although this study nicely demonstrated the effectiveness of contact tracing strategy during a period of school closure, understanding transmissibility and the implications for the reopening of schools requires reinterpretation of the data. As of April 29, 2020, a total of 37.8% of the 10–19 age group were 19 years of age (223/590) and, therefore, were not school children (*3*). A recently published study in South Korea (*4*) reported 107 primary source children (aged 0–18) had 248 household contacts and only 1 became infected, giving a secondary attack rate of 0.5%. Data from source and contact tracing in the Netherlands (*5*) also confirmed low transmissibility in children <18 years of age (0/43, 0%) compared with persons >18 years (55/566, 8.3%).

Accumulating data, including this study, suggest low transmissibility in infected children <10 years of age. However, transmissibility in the adolescent age group is unclear at this time. The 10–19 years age group includes diverse students who have completely different contact patterns from elementary school through college; thus, transmission dynamics of COVID-19 may be different. Further detailed studies on understanding transmissibility of the virus by each school level can provide helpful insights for safe reopening of schools.

## References

[1] Park YJ, Choe YJ, Park O, Park SY, Kim YM, Kim J, et al.; COVID-19 National Emergency Response Center, Epidemiology and Case Management Team. Contact tracing during coronavirus disease outbreak, South Korea, 2020. Emerg Infect Dis. 2020;26:1666–70. 10.3201/eid2608.201274 32324530 PMC7392450

[2] Mandavilli A. Older children spread the coronavirus just as much as adults, large study finds. The New York Times. 2020 Jul 18 [cited 2020 Aug 13]. https://www.nytimes.com/2020/07/18/health/coronavirus-children-schools.html

[3] Korea Centers for Disease Control and Prevention. Updates on COVID-19 in Republic of Korea, 29 April 2020 [cited 2020 Nov 13]. https://www.kdca.go.kr/board/board.es?mid=a30402000000&bid=0030&act=view&list_no=367037

[4] Kim J, Choe YJ, Lee J, Park YJ, Park O, Han MS, et al. Role of children in household transmission of COVID-19. Arch Dis Child. 2020 Aug 7 [Epub ahead of print]. 10.1136/archdischild-2020-319910 32769089

[5] National Institute for Public Health and the Environment. Children and COVID-19. 2020 Jul 20 [cited 2020 Aug 13]. https://www.rivm.nl/en/novel-coronavirus-covid-19/children-and-covid-19

